# Genetic dissection and fine mapping of a novel *dt* gene associated with determinate growth habit in sesame

**DOI:** 10.1186/s12863-018-0614-y

**Published:** 2018-06-14

**Authors:** Yanxin Zhang, Linhai Wang, Yuan Gao, Donghua Li, Jingyin Yu, Rong Zhou, Xiurong Zhang

**Affiliations:** Oil Crops Research Institute of the Chinese Academy of Agricultural Sciences, Key Laboratory of Biology and Genetic Improvement of Oil Crops, Ministry of Agriculture, No.2 Xudong 2nd Rd, Wuhan, 430062 China

**Keywords:** *Sesamum indicum* L., Determinacy, Genetic basis, Bin map, Fine mapping

## Abstract

**Background:**

As an important oil crop, growth habit of sesame (*Sesamum indicum* L.) is naturally indeterminate, which brings about asynchronous maturity of capsules and causes loss of yield.

**Results:**

The genetic basis of determinate growth habit in sesame was investigated by classical genetic analysis through multiple populations, results revealed that it was controlled by an unique recessive gene. The genotyping by sequencing (GBS) approach was employed for high-throughput SNP identification and genotyping in the F_2_ population, then a high density bin map was constructed, the map was 1086.403 cM in length, which consisted of 1184 bins (13,679 SNPs), with an average of 0.918 cM between adjacent bins. Based on bin mapping in conjunction with SSR markers analysis in targeted region, the novel sesame determinacy gene was mapped on LG09 in a genome region of 41 kb.

**Conclusions:**

This study dissected genetic basis of determinate growth habit in sesame, constructed a new high-density bin map and mapped a novel determinacy gene. Results of this study demonstrate that we employed an optimized approach to get fine-accuracy, high-resolution and high-efficiency mapping result in sesame. The findings provided important foundation for sesame determinacy gene cloning and were expected to be applied in breeding for cultivars suited to mechanized production.

## Background

Sesame (*Sesamum indicum* L.) is naturally indeterminate, the wild relatives and almost all of the cultivated sesame have indeterminate growth habit [1]. Indeterminate growth habit generates tall plant height but results in lodging during mature period, besides, it also brings about asynchronous maturity of capsules from different parts of the plant, in order to decrease immature seeds, harvesting usually performs until capsules on the upper half part mature, then basal capsules dehiscing and the seeds shattering inevitably arise, which causes loss of yield [2, 3].

The first determinate sesame mutant was discovered and reported by Ashri [1], in determinate mutants, stem growth is interrupted by the initiation of flowering on the stem apex, finally leads to the stem terminating in a flower cluster, therefore, determinate growth habit in sesame causes synchronized flowering and homogeneous capsule maturation [2], significantly reduces plant height and improves lodging resistance [3], while synchronous capsule maturation and lodging resistance were necessary to mechanized harvesting, determinate growth habit is one important prerequisite for developing sesame cultivars suited to modern planting systems combine with mechanized harvesting. Although only a few scientists engaged in determinate sesame study, it had never been ceased since first reported, except for agronomic traits observations [2–4], the oil content and fatty acid composition were also compared between determinate and indeterminate sesame [5], the determinate genotypes were found to be of higher oleic acid content and lower linoleic acid content. As to limited molecular research on determinate sesame, only an ISSR marker has been identified associated in coupling phase linkage with the gene control determinate growth habit in sesame [6].

Mutations for determinate growth habit with a short period of synchronous, uniform flowering and homogeneous maturity were important in the domestication of many plants [2], therefore, determinacy as an agronomically important trait has also been discovered and investigated in many other plants, such as soybean [7], *Arabidopsis thaliana* [8], common bean [9], tomato [10], maize [11], faba bean [12], common bean [13], chickpea [14], pigeonpea [15], cowpea [16]. Furthermore, extensive genetic and molecular studies have been implemented in these plants. Genes for determinacy of common bean, cucumber, soybean, maize and *Brassica juncea* have been located by QTL mapping [17–22], molecular makers associated with determinacy gene of faba bean, pigeonpea, common bean and soybean have been also revealed by linkage analysis or whole-genome scanning based association mapping [12, 13, 15, 23, 24]. Besides, candidate gene or homologous sequences analysis or ortholog gene sequencing approach has also been employed on common bean, soybean and pigeonpea determinacy gene isolation and characterization [25–27].

Advances in sequencing technologies have provided cost effective platforms for direct detection of high-quality SNP markers for population genotyping [28–30]. Using the sliding-window approach, adjacent SNPs with same genotype in an interval could be combined into bins that demarcate recombination locations across the whole population [31, 32]. Compared with conventional molecular markers (RFLP/SSR or single SNP), bin marker is considered to be the most informative and parsimonious set for a given population [33]. Construction of bin map benefits from this new type of bin marker which relies on automated high-throughput sequencing and genotyping technologies. The bin mapping strategy has been successfully applied to QTL fine mapping of yield associated traits in rice and sorghum [34, 35], root-knot nematode resistance and evolutionary traits in soybean [36, 37], horticultural traits in pepper [38], tassel and ear architecture, seedling root system architecture and plant architecture in maize [33, 39, 40]. In brief, the bin mapping strategy was demonstrated to be more powerful for QTL detecting than traditional methods.

In this study, the genetic basis of determinate growth habit in sesame was revealed by classical genetic analysis through multiple populations, based on a newly developed high density bin map and SSR markers, the sesame determinacy gene was fine mapped. Development of bin map was a tremendous progress in sesame, the results demonstrated that strategy optimized in this study improved accuracy, resolution and efficiency of gene mapping, the findings provided important foundation for sesame determinacy gene cloning and application in breeding for cultivars suited to mechanized production.

## Results

### Phenotypic characterization of indeterminate and determinate genotypes

**Fig. 1:**
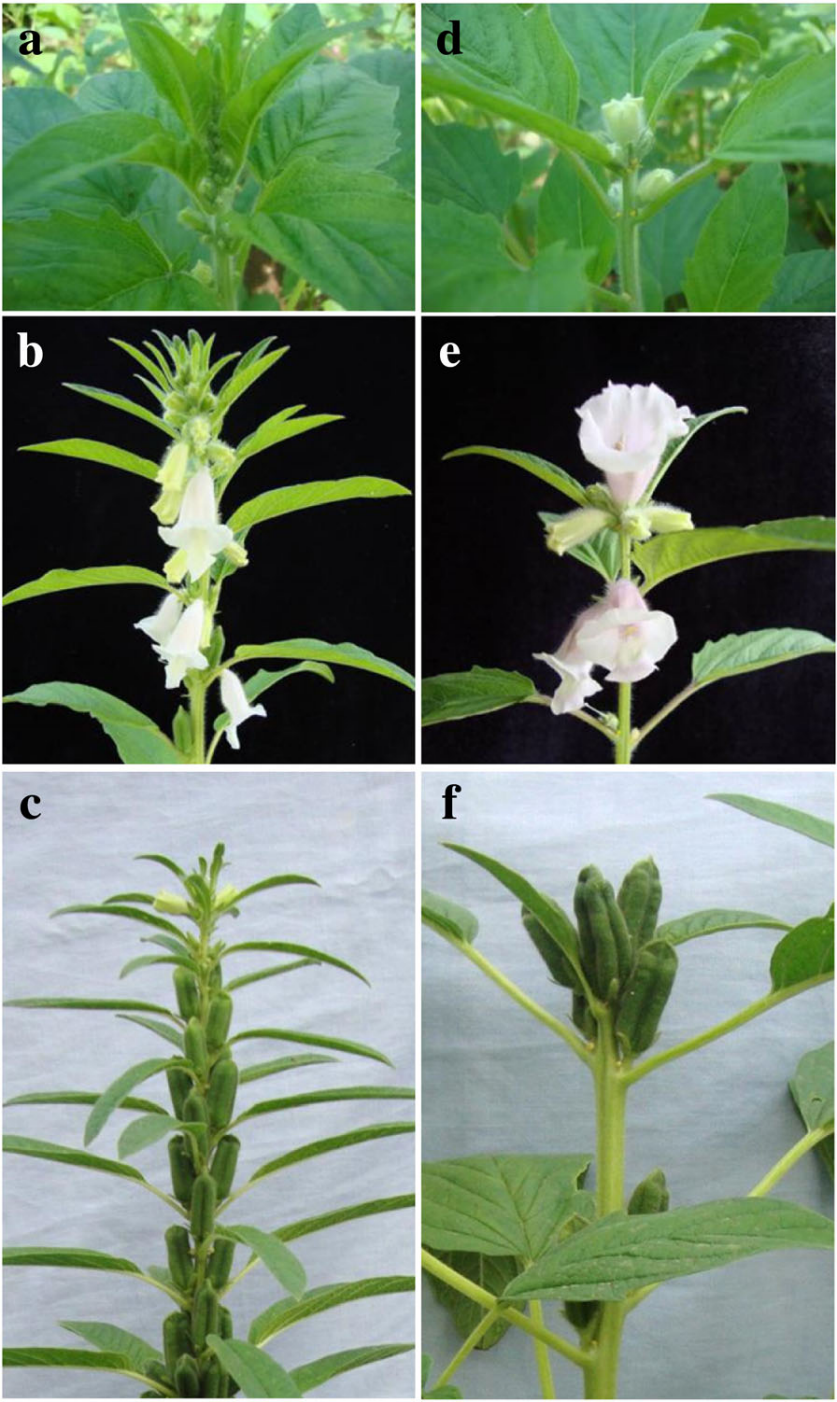
Phenotype differences of growth habit between the indeterminate and determinate genotypes. (**a**), (**b**) and (**c**) shows the flowering bud, full flowering and capsule forming stage of Zhongzhi No.13 (indeterminate genotype), respectively. (**d**), (**e**) and (**f**) shows the flowering bud, full flowering and capsule forming stage of Det-4247 (determinate genotype), respectively

Plants of Zhongzhi No.13 (indeterminate genotype) were all non-branching, their initial flowers appeared at axils of the 4th or 5th pair of leaves, inflorescences of these plants were indefinite, their apical growing point continually developed, with the elongation of rachis, flower buds differentiated successively until to the top (Fig. 1a, b). At the mature stage, number of nodes with capsules varied from 17 to 30 per plant (Fig. 1c), with an average of about 23, and the plant height was normally about 165 cm in average. Comparing with Zhongzhi No.13, phenotypic variations were displayed in Det-4247 (determinate genotype). Plants of Det-4247 were all branching, there were usually 2 primary branches from the main stem, derived with secondary branches. When the 6th or 7th pair of leaves fully developed, the stem apex meristem developed into a floral meristem, forming a terminal flower (Fig. 1d, e), then the main stem terminated, and other flowers successive appeared at the axils of second and third pairs of leaves from top, which demonstrated a typical definite inflorescence. The similar pattern was also observed on their branches. So there were only 2 or 3 nodes with capsules on the main stem and a capsule cluster was formed (Fig. 1f). As both the main stem and branches were determinate, plant height of determinate plants were normally about only 126 cm in average.

### Inheritance of growth habit

**Fig. 2:**
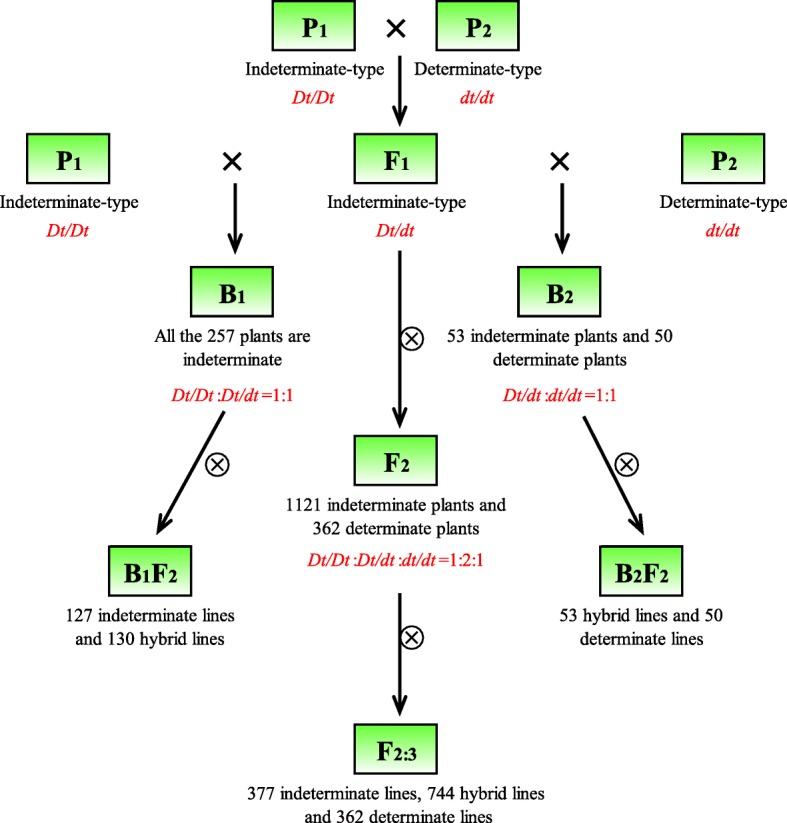
Segregation in several generations derived from the cross of Zhongzhi No.13 (P_1_) with Det-4247 (P_2_) revealed monogenic recessive inheritance pattern of determinacy

The F1 plants, which developed from the cross of Zhongzhi No.13 (indeterminate genotype) with Det-4247 (determinate genotype) were all completely indeterminate, indicating the dominance of indeterminacy. Plants of segregated generations (F_2_, F_2:3_, BC_1_ and BC_1_F_2_) showed either completely indeterminate or completely determinate phenotype, not any semi-determinate progeny was found. Segregation of indeterminate and determinate plants in all generations revealed a monogenic inheritance pattern (Fig. 2). Growth habit observation, genotype prediction and Chi-squared test revealed that genotypes in F_2_ generation segregated in a pattern of *Dt/Dt*: *Dt/dt*: *dt/dt* = 1:2:1 (*χ*^*2*^ = 3.8213, *P* = 0.8520), while genotypes in B_1_ and B_2_ generations segregated in *Dt/Dt*: *Dt/dt* = 1:1 (*χ*^*2*^ = 2.0882, *P* = 0.8516) and *Dt/dt*: *dt/dt* = 1:1 (*χ*^*2*^ = 1.4257, *P* = 0.7675) patterns, respectively (Table 1).

**Table 1:** Chi-squared test for segregation pattern in F_2_, B_1_ and B_2_ populations

Phenotype (Genotype)		Indeterminate-type (*Dt/Dt*)	Indeterminate-type (*Dt/dt*)	Determinate-type (*dt/dt*)	*χ* ^*2*^	*P*
F_2_	Plant number	377	744	362	3.8213	0.8520
	Segregation pattern	1	2	1		
B_1_	Plant number	127	130	\	2.0882	0.8516
	Segregation pattern	1	1	\		
B_2_	Plant number	\	53	50	1.4257	0.7675
	Segregation pattern	\	1	1		

### Sequencing, genotyping & linkage map developing

**Fig. 3:**
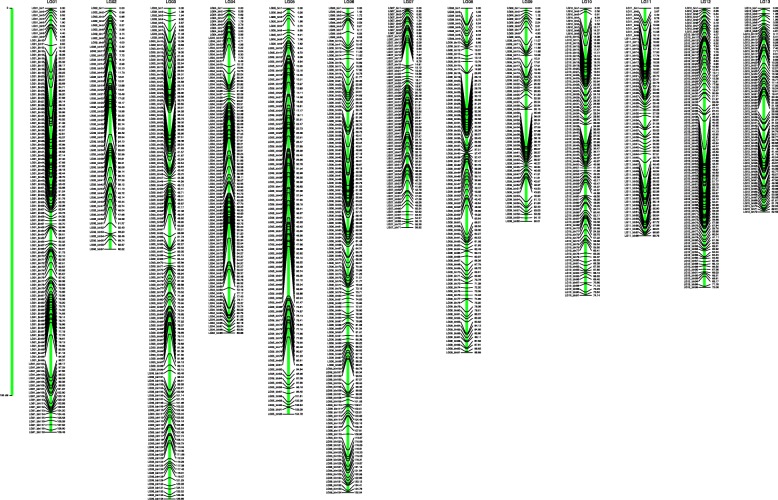
Bin map constructed for sesame in this study

Total of 232 F_2_ individuals were randomly selected for sequencing and genotyping. After library construction and high–throughput sequencing of parents and F_2_ individuals, total of 17.27 Gb raw data were generated, and a mean of 73.79 Mb high-quality sequence data were produced by each individual. Tags from the two parents were subjected to comparative analysis to detect single nucleotide polymorphism (SNP) survey. After filtering out poor SNPs, total of 13,692 polymorphic SNP markers were detected, then these SNPs were retained for genotyping of the F_2_ population. According to the 13 anchored pseudo-chromosomes, recombination loci of 232 F_2_ individuals were identified and bin genotypes of each individual were determined. After linkage analysis, 1184 bins (13,679 SNPs) were mapped onto 13 linkage groups (LGs), while the LG number was equal to the haploid chromosome number of *Sesamum indicum* L.. This linkage map was 1086.403 cM in length with an average distance of 0.918 cM between adjacent bins (Table 2, Fig. 3). The largest LG was LG04 with 134 bins, and a length of 126.655 cM, while LG09 was the smallest LG, with 72 bins, a length of 52.588 cM, and an average distance of only 0.730 cM between adjacent bins.

**Table 2:** Description on basic characteristics of the linkage map

Linkage group	SNP number	Bin number	Genetic distance (cM)	Average distance between bins (cM)
LG01	829	92	104.776	1.139
LG02	1574	96	72.061	0.751
LG03	1785	91	88.886	0.977
LG04	1149	134	126.655	0.945
LG05	514	57	62.216	1.092
LG06	420	71	72.961	1.028
LG07	1627	99	83.861	0.847
LG08	1086	91	74.139	0.815
LG09	659	72	52.588	0.730
LG10	1315	119	109.455	0.920
LG11	1757	134	125.039	0.933
LG12	467	60	55.011	0.917
LG13	497	68	58.755	0.864
Total	13,679	1184	1086.403	0.918

### Primary mapping of determinacy gene

Primary mapping of determinacy gene was performed based on acquirement of genotypic and phenotypic data of 232 F_2_ individuals. By composite interval mapping (CIM) method, and scanning in genome wide range, the gene for sesame determinant growth habit was finally located on the up end of LG09, while flanked by Bin1 and Bin2, with a LOD value of 27.4, an additive effect of − 0.8452, and a dominant effect of − 0.1064, which could explain 35.45% of the phenotypic variation (PVE). It ranged from 0 to 1.2 cM of genetic distance on LG09, while the corresponding genome region was about 1979 kb in physical length.

### Fine mapping of determinacy gene

**Fig. 4:**
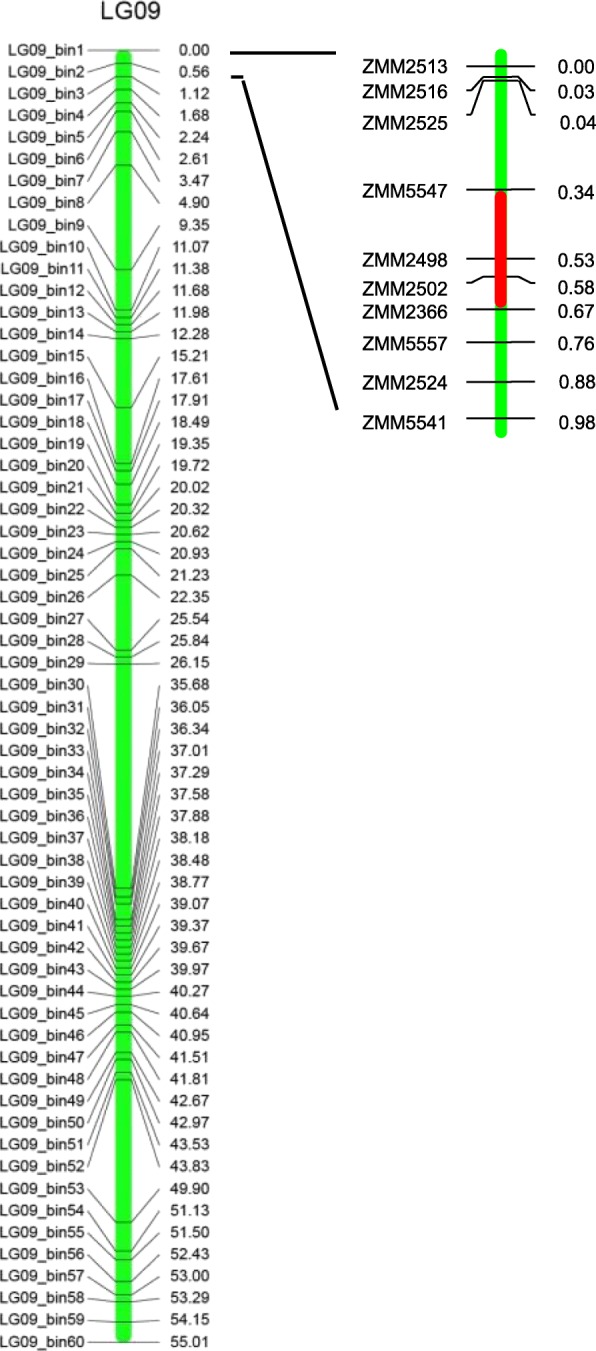
Fine mapping of sesame determinacy gene on LG09. Confidence interval was filled in red color

In order to sharpen the mapping interval of the sesame determinacy gene, we enlarged the mapping population and employed SSR markers in targeted region for genotyping. Total of 48 pairs of SSR primers were specially developed in the primary mapping genome region, and 19 of them were identified to be polymorphic between parents. These polymorphic SSR markers were subsequently used for genotyping 378 F_2_ individuals. After linkage analysis and composite interval mapping (CIM), the sesame determinacy gene was finally mapped between SSR markers ZMM5547 and ZMM2366 (Fig. 4), with a LOD value of 105.2, an additive effect of − 0.8956, and a dominant effect of − 0.0637, which could explain 66.24% of the phenotypic variation. The nearest marker ZMM2498 is only 0.022 cM from the gene. The interval of both sides was just 0.2 cM, after sequence alignment, the sesame determinacy gene was located in a 41 kb of genome region (from 53,639 bp to 94,624 bp on chr09).

## Discussion

### Inheritance of sesame determinacy gene

Determinate genotypes have been found in many species, it has been proved that determinate growth habit was controlled by a single gene in some cases, but in other studies oligogenic genes were discovered responsible for the transition of different growth habit [41]. Shannon and Meeks-Wagner [8] reported the first determinate mutant in Arabidopsis, its inflorescence meristem development was affected by TFL1 (TERMINAL FLOWER 1) gene mutation. Dt1 and Dt2, which affected the termination of apical stem growth were found in soybean by Bernard [7], and Dt1 was proved as an ortholog of Arabidopsis TFL1 gene [26]. The determinacy gene *GmTfl1* in soybean was proved to complement the functions of TFL1 in Arabidopsis [26, 41]. The determinate mutant (*det*) in pea was caused by mutations in a homologue of the Arabidopsis TFL1 gene [42]. In common bean, the gene *PvTFL1y* was co-segregated with the determinacy locus *fin* [27] and then it was also found as a functional homolog of Arabidopsis TFL1 gene [43]. Classical genetic analysis revealed that determinate genotype in this study was typical monogenic recessive. What is more important, materials used in this study possesses only two types of stem growth habits, determinate stem and indeterminate stem, there was not any intermediate type even though in the six derived segregative populations, F_2_, F_2:3_, B_1_, B_2_, B_1_F_2_ and B_2_F_2_. That’s the most distinct difference between sesame and other plants which simultaneously generated intermediary semi-determined types, such as soybean (*Glycine max*) [7].

### Development of bin map is a tremendous progress in sesame

Genetic linkage map is the most important basis of QTL mapping. Since 2009, several genetic maps have been constructed for sesame. Wei et al. [44] published the first genetic map, which was consisted of only 220 markers, but 30 linkage groups (LGs). Then this map was comparatively saturated with 653 SSR markers [45], and concentrated to 14 LGs. Employed specific length amplified fragment sequencing (SLAF-seq) strategy, Zhang et al. [46] constructed a high-density genetic map including 1233 markers distributed on 15 LGs. Wu et al. [47] developed another high-density genetic map using the restriction-site associated DNA sequencing (RAD-seq), the map included 1230 markers on 14LGs. Wang et al. [48] constructed the first bin map for sesame, which consisted of 1522 bins (11,924 SNPs) grouped into 13 LGs. Zhang et al. [49] reported a SNP map comprised of 3041 bins including 30,193 SNPs in 13 LGs. Uncu et al. [50] published an improved map of 13 LGs encompassed 432 markers (420 SNPs, 12 SSRs). In the present study, a genotyping by sequencing (GBS) approach was employed for high-throughput SNP identification and genotyping, then a high density bin map was constructed, which consisted of 1184 bins (13,679 SNPs), with an average of 0.918 cM between adjacent bins, while the bin marker density was similar to the map density of one bin per 0.98 cM described by Zhang et al. [49]. Furthermore, the number of LGs in our newly developed map is just equal to the haploid chromosome number of *Sesamum indicum* L., these will also be its obvious advantages in future application.

### Strategy optimized in this study improved accuracy, resolution and efficiency of gene mapping

Resolution of QTL mapping depends on the recombination frequency, the coverage of genetic markers, and the population size. Since the lack of reliable markers and the less development of genetic maps in sesame, only few articles reported QTL or gene mapping by linkage analysis. The first QTL analysis of sesame important agronomy traits focused on seed coat color [45], and 4 QTL were detected based on a linkage map constructed by conventional markers (SSR, AFLP, and RSAMPL), while the QTL intervals ranged from 1.0 cM to 23.4 cM. Wu et al. [47] detected 30 QTL for 7 grain yield-related traits based on a high-density genetic map constructed by SNP markers, and the QTL regions varied from 0.1 cM to 24.6 cM. Wang et al. [48] identified 41 QTL for sesame plant height and 9 for seed coat color on their bin map, and the most accurate locus was located on Chr4 in a 199.9 kb space. In our study, after primary mapping on bin map in whole genome scale, we tended to employ SSR markers to densify the targeted region and enhance accuracy for fine mapping, as genotyping by SSR was a mature technology utilized in our lab, which was highly specific, independent of sequencing, repeatable, and low-cost. More importantly, masses of SSR markers had been developed from the whole genome survey in our previously study. Combining bin mapping with targeted region densified by SSR in fine mapping, the candidate block of the sesame determinacy gene was sharped into a genome region of just 41 kb, and only 10 genes was subsequently predicted in this region, which paved the way for obtaining and cloning candidate gene. The fine-accuracy, high-resolution and high-efficiency of mapping result of this study showed the advantages of strategy employed, and also provided valuable technique foundation for further QTL mapping of other key agronomic traits in sesame.

### The determinacy gene discovered in this study was a novel *dt* gene controlling determinate growth habit

The *SiDt* gene associated with determinate growth habit in sesame has been reported by Zhang et al. [49], which was discovered from an EMS mutant Yuzhi DS899, in their study, the locus (QDt1) was located to LG8, with an inheritance interval of 18.0 cM–19.2 cM. In contrast, the determinacy gene mapped in this study was discovered from a natural germplasm. In order to verify the identity or dissimilarity between the *SiDt* gene and the newly discovered determinacy gene here, we performed gene alignment of the *SiDt* gene sequence in Zhongzhi No.13 reference genome [48], and the gene SIN_1010554 with 100% identity to the *SiDt* gene sequence was identified, then the position of SIN_1010554 was further checked, which was located in the middle part of chr09, from 649,613 bp to 651,421 bp, while the determinacy gene mapped here was located in a different genome region, which near the up end of chr09, from 53,639 bp to 94,624 bp. So the results of position compare indicated that they were absolutely disparate genes controlling different determinate genotypes. In addition, positions of all the 7 homologs of *SiDt* gene were also investigated, and results showed that none of them was within or around the region of our novel *dt* gene. In summary, the determinacy gene discovered in our study was a novel *dt* gene controlling determinate growth habit in sesame.

### Application prospects of sesame determinacy gene

Genes associated with determinate growth habit have been mapped in several species, such as common bean *(Phaseolus vulgaris)*, pigeonpea (*Cajanus* spp.), Indian mustard (*Brassica juncea*), soybean (*Glycine max*), etc. In common bean, using bulked segregant analysis (BSA) and linkage analysis, 2 markers were identified to be linked to the *fin* gene (3.3 to 26.9 cM) [13]. In pigeonpea, association mapping showed a significant association (*P* ≤ 0.01) of determinacy with 19 SNP explaining 8.05–8.58% phenotypic variation [15], QTL analysis highlighted CcTFL1 as a likely candidate gene, which was located in a genomic region of 24 cM [25]. Kaur and Banga [20] mapped Indian mustard determinate growth habit gene (*Sdt1*), which was flanked by 2 SSR markers at distances of 15.9 cM and 14.0 cM. Vicente et al. [23] mapped the molecular markers of genes Dt1 and Dt2 in soybean, and sat_064 marker associated with Dt2 exhibited a recombination frequency of 19.4%. Nevertheless, most of these QTL/gene from above species were primarily located in a large genome scale, since the QTL regions were not small enough, it was far from candidate gene predication or map-based gene cloning. What was encouraging, based on our fine mapping result, the determinacy gene is cloning and verifying in our laboratory (unpublished study), and will be applied in sesame germplasm innovation or molecular breeding in future through genetically modification or gene pyramiding methods. Ultimately, the newly developed sesame cultivars with determinate growth habit, synchronizing flowering, early maturation and lodging resistance will be popular in the mechanized production, and their application prospects will be promising.

## Conclusions

We dissect the genetic basis of determinate growth habit in sesame, constructed a new high-density bin map and mapped a novel determinacy gene. Results of this study demonstrate that bin mapping in conjunction with SSR markers analysis in targeted region was an optimized approach to get fine-accuracy, high-resolution and high-efficiency mapping result in sesame. The findings provided important foundation for sesame determinacy gene cloning and were expected to be applied in breeding for cultivars suited to mechanized production.

## Methods

### Parental genotypes, population development and phenotyping

The female, Zhongzhi No.13, is a widely cultivated sesame variety in China, with indeterminate growth habit. The donor parent, Det-4247, on the other hand, is a special germplasm accession queried from the national mid-term genebank for oil crops, with typical determinate growth habit. F1 plants were developed from the crossing of Zhongzhi No.13 (indeterminate genotype) with Det-4247 (determinate genotype). F_2_ population derived from the self-crossing of F_1_, which consisted of 1483 individuals. Zhongzhi No.13 crossed with F_1_ and generated B_1_ population (257 individuals), and F_1_ crossed with Det-4247 then generated B_2_ population (103 individuals). The self-crossing of F_2_, B_1_, and B_2_ generated three populations of F_2:3_ (1483 lines), B_1_F_2_ (257 lines), and B_2_F_2_ (103 lines), respectively. The determinate or indeterminate growth habit of all above materials were investigated during flowering stage and verified in maturation stage, according to IPGRI and NBPGR [51].

### DNA extraction, enzyme digestion and sequencing

Genomic DNA was extracted from young healthy leaves of two parents and each F_2_ individual, according to the cetyltrimethylammonium bromide (CTAB) method [52]. DNA concentration and quality were estimated with a Qubit Fluorometer (using Quant-iT™ dsDNA HS Assay Kit), and also by electrophoresis in 0.8% agarose gels with a lambda DNA standard. DNA with quality conformance was then digested with the *Taq* I restriction enzyme (Takara, Dalian, China), and adapters containing a multiplex identifier (MID) were added to the samples [48]. DNA fragments that were 350–600 bp in length were selected by gel electrophoresis from the library samples. Then the libraries were enriched by PCR amplification, quantified on an Agilent 2100 Bioanalyzer (Agilent Technologies, Santa Clara, CA, USA), and finally sequenced on an Illumina HiSeq2000 instrument (San Diego, CA, USA).

### SNP calling and genotyping

Raw reads with low quality in which 10% of the nucleotides had a quality value less than Q30 (0.1% sequencing error) were discarded. The clean pair-end reads were further trimmed to the RAD tags with a uniform length of 94 nucleotides. All of the high quality reads were aligned with reference genome of Zhongzhi No.13 (BioProject PRJNA301193 in NCBI) using BWA software [53]. The “mpileup” function in SAMtools software [54] was employed to detect SNPs between parental lines using reads with mapping quality values greater than or equal to 20. The detected SNPs were used for genotyping each individual in F_2_ population, only individuals with more than 20,000 SNPs were selected to do the following analysis.

### Construction of bin map

The bin map was constructed according to the sliding window approach developed by Huang et al. [31]. The *Dt/Dt*: *Dt/dt*: *dt/dt* segregation ratios of SNP markers were calculated, and their fitness to 1:2:1 were evaluated by the chi-square test, significantly (*P* < 0.01) distorted markers were discarded. The consecutive genotypic SNPs were scanned with a sliding window size of 15 SNPs and a step size of 1. Based on the recombinant breakpoints, adjacent windows with the same genotype were combined into blocks, which were considered to be bins [33, 48]. The bin markers were analyzed using JoinMap 4 software [55] with a logarithm of minimum odds (LOD) of 4.0, the Kosambi mapping function [56] was used to transform the recombination frequency into map distance in centiMorgans (cM), and then the bin map was constructed.

### Primary mapping and fine mapping of determinacy gene

Based on the bin map, genotypes of bin markers, and growth habit phenotypes of F_2_ individuals, QTL mapping was performed using composite interval mapping (CIM) method [57], employed WinQTLCart 2.5 software (http://statgen.ncsu.edu/qtlcart/WQTLCart.htm). The likelihood ratio statistic was computed for each bin. The LOD thresholds were obtained based on permutation test (1000 permutations, *P* = 0.05). After primary mapping, the major QTL was detected, and the QTL interval and corresponding genome region were uncovered. In order to mapping the determinacy gene within a more accurate region, SSR markers were selected specially from the primary mapping genome region, based on the 23,438 SSR markers previously developed from the whole genome survey [58, 59], selected SSR markers were subsequently screened for polymorphism between parents, and then used to genotyping a larger F_2_ population for fine mapping of determinacy gene. The corresponding physical distance of the mapping interval was obtained by aligning with the Zhongzhi No.13 reference genome (BioProject PRJNA301193 in NCBI).

## Abbreviations

BSA: Bulked Segregant Analysis
CIM: Composite Interval Mapping
cM: centiMorgans
CTAB: Cetyltrimethylammonium bromide
GBS: Genotyping by Sequencing
LG: Linkage Groups
LOD: Logarithm of minimum odds
MID: Multiplex Identifier
PVE: Phenotypic Variation
RAD-seq: Restriction-site Associated DNA sequencing
SLAF-seq: Specific Length Amplified Fragment sequencing
SNP: Single Nucleotide Polymorphism
